# Up to Date Review of Nature-Inspired Superhydrophobic Textiles: Fabrication and Applications

**DOI:** 10.3390/ma16217015

**Published:** 2023-11-02

**Authors:** Haipei Ge, Yu Liu, Fujuan Liu

**Affiliations:** National Engineering Laboratory for Modern Silk, College of Textile and Clothing Engineering, Soochow University, 199 Ren-Ai Road, Suzhou 215123, China; gehaipei2023@163.com

**Keywords:** superhydrophobic surface, biomimetic textiles, lotus leaf, theoretical modeling

## Abstract

In recent years, with the rapid development of the economy and great progress in science and technology, people have become increasingly concerned about their quality of life and physical health. In order to pursue a higher life, various functional and biomimetic textiles have emerged one after another and have been sought after by people. There are many animal and plant surfaces with special wettability in nature, and their unique “micro-nano structures” and low surface energy have attracted extensive attention from researchers. Researchers have prepared various textiles with superhydrophobic features by mimicking these unique structures. This review introduces the typical organisms with superhydrophobicity in nature, using lotus, water strider, and cicada as examples, and describes their morphological features and excellent superhydrophobicity. The theoretical model, commonly used raw materials, and modification technology of superhydrophobic surfaces are analyzed. In addition, the application areas and the current study status of superhydrophobic surfaces for textiles are also summarized. Finally, the development prospects for superhydrophobic textiles based on bionic technology are discussed.

## 1. Introduction

**Figure 1:**
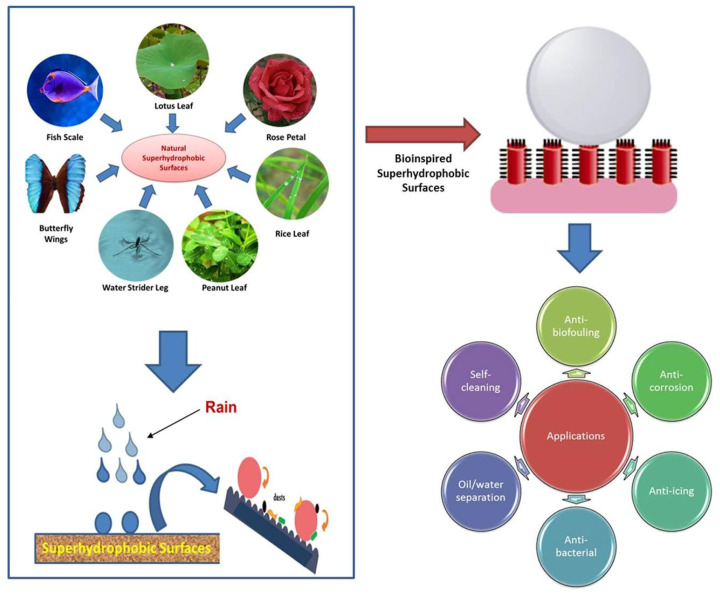
Inspiration, theory, and applications of superhydrophobic surfaces [2].

The surfaces of superhydrophobic fabrics are highly resistant to water, making it difficult for water droplets to wet them. Superhydrophobic fabric surfaces manifest water contact angles above 150° and roll-off angles below 10° [1,2,3,4,5], which is also referred to as the “lotus effect” [6,7]. In recent years, superhydrophobic surface fabrics, due to their excellent water repellency, stain resistance, self-cleaning, and reduction in fluid resistance (Figure 1) [2,3,8,9], have exhibited great development potential in many fields such as self-cleaning, biomedical applications, functional textiles, environmental remediation, and more. The reason why superhydrophobic fabrics can improve the durability and cleanability of fabrics is that superhydrophobic surfaces exclude water and other liquids, allowing water droplets to carry away surface contaminants as they rolling over the surface [10,11]. Therefore, superhydrophobic fabrics have attracted widespread attention and are increasingly becoming a new hot spot for textile research.

Organo-silicone and organo-fluorine hydrophobic finishes can significantly reduce the surface tension of fabrics, thereby effectively improving their hydrophobicity. However, there are still some limitations, such as fabric static electricity, the high cost, and the gap with the required superhydrophobicity standard [12,13]. Therefore, research has turned to animals and plants in nature. Many organisms in nature are superhydrophobic, and the lotus leaf is a typical example. The surface of the lotus leaf is covered with “micro-nano” structures. Due to the air being filled with these “micro-nano” structures, water droplets can only form a “point contact” with the lotus leaf, and cannot completely wet its surface. This indicates that the lotus leaf has superhydrophobicity and a low viscosity. Based on this property, various methods have been used to mimic the structure of the lotus leaf to create artificial superhydrophobic surfaces with contact angles greater than 150° [14]. For example, Klicova et al. [15] developed a bionic layered nanofiber surface inspired by the superhydrophobic structure of the lotus leaf to prevent tissue adhesion. In addition, it has been also observed that water striders have many nanoscale grooved structures on the setae on their legs, which enable them to walk on water surfaces [16,17]. The dorsal and ventral sides of the cicadas wings have a columnar structure [18], butterfly wings exhibit a directional adhesion function [19], and the mosquito compound eye possesses a remarkable special micro-nano hierarchical structure and low surface energy [20], which have endowed them with superior superhydrophobicity. Koh et al. [21] were inspired by the jumping ability of the water strider on the surface of water anddeveloped a micro-robot that could be used for environmental monitoring and military surveys. Inspired by the non-fogging property of the wing scales of the Mediterranean macro blue butterfly, Han et al. [22] utilized optimized biotemplate-assisted wet chemistry to design and prepare a glass-based multi-scale hierarchical tower-like structure (MHPS) micro-robot without any post-processing. In summary, for superhydrophobic materials, the following two conditions need to be fulfilled: On the one hand, constructing surface roughness to form micro-nano structures; on the other hand, modifying fabric surfaces using low-surface-energy materials [23,24].

The common raw materials for obtaining superhydrophobic rough surfaces contain inorganic particles, such as ${\mathrm{SiO}}_{2}$ and ${\mathrm{TiO}}_{2}$, which provide strong conditions for the preparation of superhydrophobic surfaces. However, the high price and the inability to guarantee the mechanical stability of the materials limit their end use. Therefore, researchers have turned their attention towards developing cellulose-based and lignin-based superhydrophobic surfaces, which are environmentally friendly natural fiber materials with excellent properties for greatly improving the wear resistance and mechanical stability. Due to the superior features of self-cleaning and a reduced fluid viscosity, superhydrophobic surfaces have broad prospects in the fields of self-cleaning, biomedical applications, functional textiles, and environmental remediation.

The preparation of superhydrophobic surfaces requires a combination of a rough surface morphology and low surface energy. Several chemical and physical methods have been developed to fabricate such surfaces. The preparation methods can be categorized into single-step and multi-step processes [3]. The single-step process means that the surface is roughened and chemically modified using a one-step process. Common one-step methods include sol-gel, electrospinning, templating, and vapor phase deposition. In comparison, the multi-step process involves roughening the surface firstly and then modifying it by adding low-surface-energy coating materials.

This review presents an overview of several common organisms that exhibit superhydrophobic features, such as the lotus, water strider, and cicada, etc. The basic models and principles for surface wettability, including the Young’s, Wenzel, and Cassie models, are then introduced. Additionally, the work also discusses the commonly used raw materials and preparation methods for modifying textile surfaces to become superhydrophobic. In the end, the possible applications for superhydrophobic surfaces in the textile industry are outlined with the aim of deepening our understanding of bionic technology and inspiring new ideas for its implementation.

## 2. Common Organisms with Superhydrophobic Features

After 100 million years of reproduction and evolution on the earth, many organisms have undergone changes to adapt to the survival environment, gradually forming various regular rough structures on their surfaces. Examples include lotus leaves, water striders, cicadas, butterflies, and rose petals, all of which have excellent superhydrophobicity.

### 2.1. Lotus Effect

**Figure 2:**
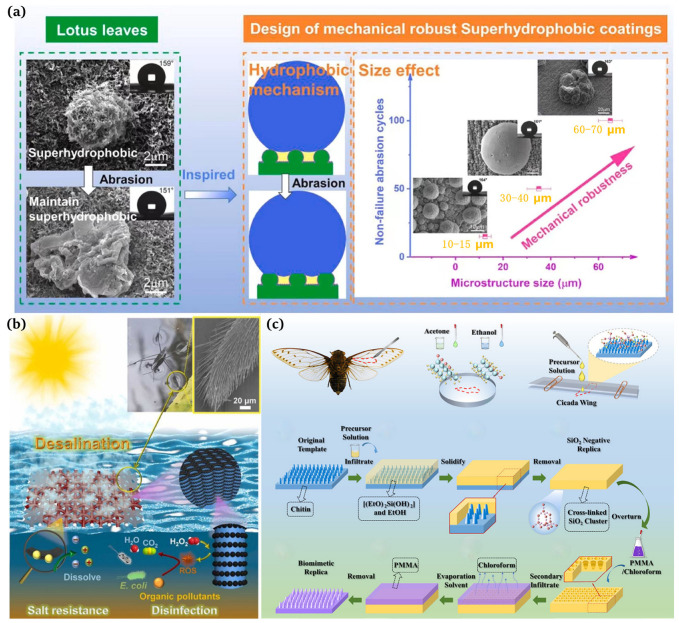
(**a**) Design of robust superhydrophobic copper coatings inspired by the lotus leaf effect [29]; (**b**) leg structure of a water strider and the function mechanism of a floating solar evaporator with a high salt tolerance influenced by the water strider [30]; (**c**) optical photographs of cicadas and the preparation of the self-cleaning broadband anti-reflection film [34].

“Out of the silt and not stained” is a well-known poem praising the lotus flower, which explains that the lotus leaf has a self-cleaning function and is attributed to the micron-sized papillary structures and wax on its surface [25,26,27]. Barthlott et al. [25] found that the superhydrophobicity of lotus leaves was due to the presence of long-chain olefins with a low surface energy. In addition, it was observed using scanning electron microscopy (SEM, Auriga 60, Zeiss AG, Jena, Germany) that the surface of the lotus leaf has a micro-scale papillae structure, and there are dendritic nanoscale projections composed of epidermal hydrophobic waxy tubules [28]. The presence of these “micro-nano” structures and low-surface-energy waxes results in a high water contact angle and a low rolling angle of 164° and 3°, respectively, thus achieving high hydrophobicity and self-cleaning functions. Therefore, we understood that a micron-nano fine binary structure exists on the rough surface of the lotus leaf. One layer of the structure consists of micro-sized papillae of approx. 11 µm in diameter and 13 µm in height. The top of the papillae has wax secreted by the epidermis, similar to the surface morphology of hair strands or velvet. Inspired by lotus leaves, Yang et al. [29] developed superhydrophobic copper with mechanical elasticity that exhibited outstanding corrosion resistance and self-cleaning properties (Figure 2a).

### 2.2. Water Strider Phenomenon

Water striders are representative of animals with superhydrophobic structures in nature. They have three legs, and a distal segment of each leg is composed of the femur, tibia, and tarsus bones. The legs are covered with uniformly inclined setae, each with grooves. Drawing inspiration from water striders, Wang et al. [30] documented a solar evaporator with a high salt tolerance that is capable of purifying polluted seawater for desalination (Figure 2b). Motivated by water striders, Luo et al. [31] presented a novel solar evaporator comprising a hydrophobic frame, a hydrophilic solar absorber, and a heat insulation layer. Based on the surface tension force created by the hydrophobic frame, the solar evaporator can stably float just below the water surface, allowing for the confinement of a thin water film on the solar absorber for salt resistance and efficient energy applications. In particular, the suspension depth was maintained under almost unaltered conditions in brines of different salinities, resulting in high evaporation rates (1.45 kg m^−2^h^−1^ and 1.35 kg m^−2^h^−1^) at high salt concentrations (15 wt% and 20 wt% NaCl) under one sun illumination.

### 2.3. Superhydrophobicity of Cicadas

Wang et al. [32] measured the water contact angle of cicada wings with the help of a water droplet gauge (KRUSS, GER, DSA25) at 138.8° and observed a thickness of 6.25 um using a field emission electron microscope (FESEM, JEOL, Akishima, Japan, JSM6700F). These nanopillars had a diameter of approx. 80 nm, a height of approx. 200 nm, and a spacing of approx. 80 nm. It was these regularly arranged nanopillars that increased the surface roughness of the cicada wings and made them superhydrophobic, ensuring that their surfaces did not attract airborne dust, rain, or dew [33,34]. Han et al. [34] prepared biomimetic superhydrophobic membranes with anti-reflective and self-cleaning properties using silica (${\mathrm{SiO}}_{2}$) templates (Figure 2c).

### 2.4. Other Superhydrophobic Phenomena in Nature

In addition to lotus leaves, water striders, and cicadas, there are many other organisms in nature that are superhydrophobic, such as rice leaves, rose petals, dragonflies, and butterfly dragonflies. Wang et al. [35] proposed a technique for producing superhydrophobic magnetorheological elastomers (MREs) with a magnetic responsiveness, which could instantly and reversibly switch between the ‘lotus effect’ and the ‘rose petal effect’.

## 3. Basic Models and Principles of Surface Wettability

### 3.1. Principles of the Contact Angle

**Figure 3:**
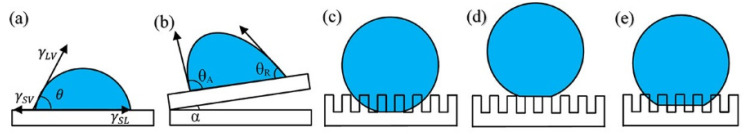
Various states of water droplets on a solid surface [40]: (**a**) Young’s state; (**b**) contact angle hysteresis; (**c**) Wenzel’s state; (**d**) Cassie–Baxter state; (**e**) transition between the Wenzel and CB states.

As shown in Figure 3a, wettability is conventionally assessed using the contact angle, which is formed by the tangent to the liquid/vapor interface and the solid surface at the three-phase contact line. The contact angle is commonly used to measure the wetting properties of a liquid on a solid surface. In 1805, to estimate the contact angle of the liquid on a homogeneous and absolutely smooth surface, Thomas Young formulated the following model.

(1) $${\cos\theta }_{w}=\frac{\gamma_{\mathrm{SV}}-{\;\gamma }_{\mathrm{SL}}}{\gamma_{\mathrm{LV}}}$$

where $\theta_{w}$ is the apparent contact angle and $\gamma_{sl}$, $\gamma_{sv}$, $\gamma_{lv}\;$ are the solid–liquid interface, solid–air interface, and liquid–air interface, respectively.

According to Young’s formula [36], it can be seen that the smaller the contact angle, the better the wettability. Typically, the contact angle is generally bounded by 90°. When the contact angle θ>90°, the solid is hydrophobic. When the contact angle θ>90°, the solid is hydrophilic. According to Young’s equation, for small drops, the contact angle ($\theta_{w}$) is adequate to determine the shape of the liquid drop and its wettability. The necessary condition for a drop to spread is ($\gamma_{\mathrm{SV}}-\gamma_{\mathrm{SL}}$) > $\gamma_{\mathrm{LV}}$, i.e., the energy to create the solid–vapor interface should be more than that of the liquid–vapor interface. If this condition is not fulfilled, the drop will not spread, resulting in a contact angle ($\theta_{w}$) [37]. Nevertheless, Young’s equation is an idealized assumption that only applies to solids with smooth, non-deforming, impermeable surfaces. In actuality, discrepancies exist between the surface characterization contact angle and the inherent angle due to varying solids’ surface roughness. As a result, Young’s equation cannot be entirely valid.

### 3.2. Theoretical Models of Superhydrophobicity

To characterize the wettability of solid surfaces more accurately, Wenzel and Cassie et al. introduced the concept of roughness *r* and proposed the Wenzel [38] and Cassie [39] models.

#### 3.2.1. The Wenzel Model

Briefly, the Wenzel model (Figure 3c) assumes that for a solid with a rough surface, a liquid can completely immerse into the rough grooves and fill them, resulting in a larger solid–liquid contact area of the actual solid than the solid–liquid contact area seen on the solid surface. In other words, the solid can increase the surface area by increasing the surface roughness. Therefore, if the solid has hydrophobic properties, the surface energy can be reduced more to enhance the hydrophobicity of the solid, as shown in Equation (2).

(2) $${\cos\theta }_{w}=r\cos\theta =r\frac{\gamma_{\mathrm{SV}}-\gamma_{\mathrm{SL}}}{\gamma_{\mathrm{LV}}}$$

where *θ* is the intrinsic contact angle and *r* is the roughness (the ratio of the actual geometric area of solid–liquid contact to the projected area of the horizontal plane, *r* ≥ 1).

According to the Wenzel equation, when θ < 90°, the solid surface is hydrophilic. When 0 < cosθ < $\cos\theta_{w}$, that is, when $\theta >\theta_{w}$, rough surfaces can increase their hydrophilicity. When θ>90°, 0 > cosθ > $\cos\theta_{w}$, namely 0 < $\theta_{w}$, the roughness can enhance the hydrophobic nature of the surface. Therefore, if the superhydrophobic surface is obtained under the Wenzel model, then *θ* must be larger than 90°.

#### 3.2.2. The Cassie Model

The Cassie–Baxter (Figure 3d) model shows that when the contact angle is greater than 90°, the droplets do not fully fill the rough grooves. If the solid film is rough, it is air-doped, that is, there is a large amount of air in the rough structural body that prevents the droplets from wetting the solid surface and forming a solid-liquid-gas three-phase contact surface. At this point, the apparent contact angle of the solid is written as follows.

(3) $${\cos\theta }_{c}=f_{1}{\cos\theta }_{1}+{\mathrm{rf}}_{2}{\cos\theta }_{2}$$

If we use ${\;f}_{s}\;$ to represent the liquid–solid interface by the liquid wetting of the solid surface fraction, then the liquid–gas interface area fraction is 1 − $f_{s}$. When the gas–liquid contact angle ${\;\theta }_{2}$ is 180°, the equation can be expressed as follows.

(4) $${\cos\theta }_{c}=f_{s}\cos\theta +(1-f_{s}){\cos180\text{°}=f}_{s}\cos\theta +f_{s}-1$$

(5) $$f_{s}=\frac{S_{P}}{S_{A}}$$

where $\theta_{c}\;$ is the apparent contact angle; $f_{1}\;$ is the area fraction of the liquid–solid interface; $f_{2}\;$ is the area fraction of the gas–liquid interface; $\theta_{1}\;$ is the intrinsic contact angle of the liquid–solid interface; $\theta_{2}$ is the intrinsic contact angle of the gas–liquid interface; $\theta_{c}$ is the apparent contact angle of the composite surface; *θ* is the intrinsic contact angle; $f_{s}\;$ is the area fraction of the solid in the composite contact surface; $S_{P}$ is the area of the solid protruding from composite contact surface; and $S_{A}$ is the apparent area of the composite contact surface.

According to the Cassie equation, with an increase in the solid surface roughness and a decrease in $\;f_{s}\;$, ${\cos\theta }_{c}\;$ decreases and $\theta_{c}\;$ increases, resulting in a more hydrophobic solid surface. When the roughness is large enough, $f_{s}\;$ is infinitely close to 0, ${\;\cos\theta }_{c}$ is infinitely close to −1, and $\theta_{c}$ is close to 180°, then the solid surface has superhydrophobicity. Therefore, under the Cassie model, no matter how big *θ* is, a superhydrophobic surface may be obtained.

## 4. Common Raw Materials and the Modification Preparation of Superhydrophobic Surfaces

### 4.1. Common Raw Materials of Bionic Superhydrophobic Surfaces

Currently, inorganic particles are typically used as raw materials for the construction of rough surfaces with a good hydrophobicity. However, such rough surfaces are known to have unstable mechanical properties. To overcome their poor mechanical properties, cellulose and lignin-based raw materials have been introduced. These natural materials possess unique physical and chemical properties that help maintain the performance stability of the surface roughness to a certain extent.

#### 4.1.1. Inorganic Particles

**Figure 4:**
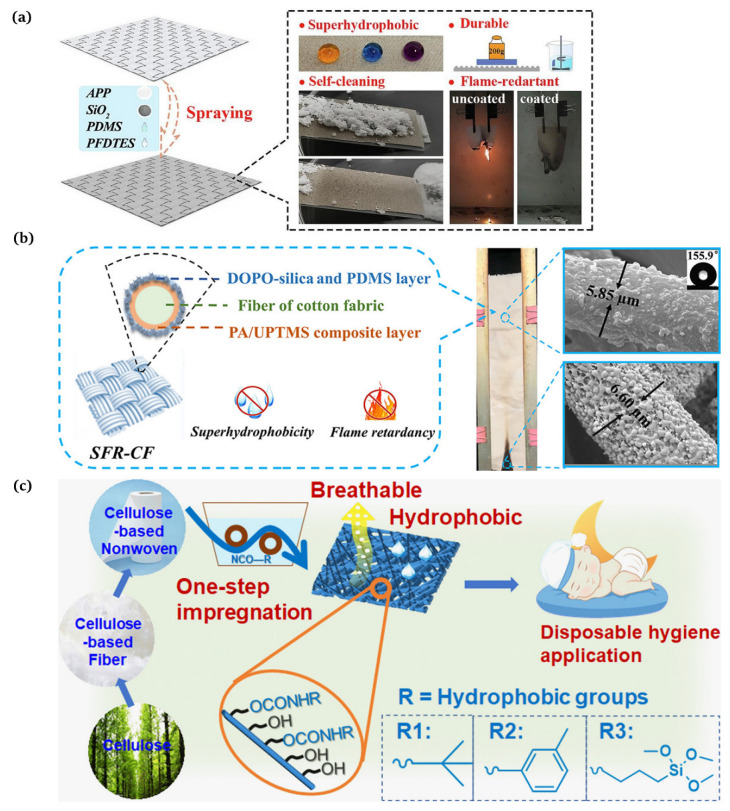
(**a**) Preparation of superhydrophobic flame retardant coatings on PET fabrics [45]; (**b**) manufacture of a superhydrophobic phosphorus–nitrogen flame retardant cotton fabric [46]; (**c**) production of hydrophobic and breathable cellulose non-wovens for disposable hygiene [56].

The substances applied to build surface roughness structures are generally inorganic particles, including ${\mathrm{SiO}}_{2}$ [41], ${\mathrm{TiO}}_{2}$ [42], and ${\mathrm{ZnO}}_{2}$ [43]. For instance, Liu et al. [44] proposed a method to design a robust superhydrophobic coating with an excellent performance via a straightforward and expeditious method. The modified silica nanoparticles were firstly sprayed onto the hydrophobic polyurethane coating and then curried under a UV lamp for 3 min to obtain a superhydrophobic surface with a water contact angle of approx. 160° and a sliding angle of 2°. He et al. [45] demonstrated a strategy to produce fluorinated PDMS through the cross-link reaction of fluorosilane and polydimethylsiloxane (PDMS, Dow Corning Ltd., Midland, MI, USA, Sylgard 184), uniformly blended with the combustion product of silicone rubber (mainly composed of ${\mathrm{SiO}}_{2}$) and ammonium polyphosphate (APP, Jianxi Nanomaterials Technology Research and Development Co., Ltd., Nanchang, China). The mixture was coated onto the PET fabric surface to form a superhydrophobic flame retardant layer (Figure 4a). Chen et al. [46] described a new condensation techinque to acquire superhydrophobic and flame resistant CF, which exhibited a high water contact angle of 155.9° and prime anti-adhesion and self-cleaning properties. CF was first coated with phytic acid (PA, Aladdin Reagent Co., Ltd., Shanghai, China) in conjunction with 1-[3-(trimethoxysilyl) propyl]urea (UPTMS, Aladdin Reagent Co., Ltd., Shanghai, China) to provide phosphorus–nitrogen flame retardancy. Subsequently, the silica particles were grafted using 9,10-dihydro-9-oxa-10-phosphaphenanthrene-10-oxide (DOPO, Aladdin Reagent Co., Ltd., Shanghai, China) to generate the surface roughness and further enhance the flame retardancy. Low surface energy polydimethylsiloxane (PDMS, USA, Sylgard 186A) was coated onto the CF surface, and the superhydrophobic and flame retardant CF (SFR-CF) was obtained after thermal curing (Figure 4b).

#### 4.1.2. Cellulose-Based Superhydrophobic Textiles

Cellulose, the most abundant biopolymer, is the main raw material for cotton [47]. It exhibits several encouraging characteristics, such as a mechanical robustness, hydrophilicity, biocompatibility, and biodegradability [48]. The surface diffusion and absorption of water by cotton fabrics and paper can be enhanced via the capillary action generated by cellulose fibers. Certain substances, such as silicone materials, fluorinated materials, and organic aliphatic long-chain molecules with a low surface energy, can be applied to lessen the surface energy of rough surfaces. Enhancing the roughness is paramount when designing superhydrophobic surfaces [49]. The two fundamental principles are also applicable to superhydrophobic coatings on cellulose-based materials [50]. In recent years, the fabrication of cellulose-based superhydrophobic materials has been divided into two aspects: on the one hand, superhydrophobic modification of fiber products based on writing paper [51], filter paper [52], cotton [53], and others using laser etching, templating, chemical deposition, and chemical etching; on the other hand, modifications based on cellulose [54] (e.g., cellulose nanobases, cellulose nanocrystals, etc.) using graft polymerization, the plasma method, and sol-gel. Cellulose-based materials are the favored choice for bedding, sportswear, and intimate apparel thanks to their manifold benefits [55]. Liu et al. [56] reported a facile one-step process to convert the completely hydrophilic cellulose non-woven substrate into highly hydrophobic fabrics (water contact angle of 130°–135°) through the preservation of good air permeability (variation ± 6% after modification compared to the original 1337 mm.s^−1^) (Figure 4c). To address the issues of the low water stability and physical strength associated with the inherent hydrophilicity of the raw material cellulose, Qin et al. [57] presented a novel, all natural superhydrophobic straw composed of a cellulose nanofiber and stearic acid composite. Stearic acid, a saturated fatty acid derived from plant and animal oils, was utilized in the CFS’s production. Drawing on the unique hydrophobicity of sugarcane cane peel, green straws demonstrated exceptional mechanical properties (tensile strength up to 67.15 MPa) and superhydrophobicity (water contact angle up to 153°) by precisely controlling the conditions of the stearic acid hydrophobic modification through solvent vaporization. In addition, the composite straws exhibited a lower water absorption and a superior wet tensile strength when compared to conventional paper straws. Furthermore, the composite straws, produced without the use of chemical binders, avoided the shortcomings of non-renewable products, which aligns with the goal of global sustainable development, and presented a novel advancement for cellulose-based materials. The degradability and the unique physical, chemical, and mechanical properties of cellulose make it the best candidate for superhydrophobic raw materials [58].

#### 4.1.3. Lignin-Based Superhydrophobic Textiles

Lignin belongs to a natural polymer consisting of three main phenylpropenoid units that are rich in aromatic ring structures and aliphatic, aromatic hydroxyl and quinone reactive groups [59,60,61]. Its low content of reactive hydroxyl groups provides an excellent UV resistance, preventing the substrate from aging to a certain extent, and has natural advantages in the development and applications of superhydrophobic surfaces [62]. Lignin can be divided into four different types of technical lignins, depending on the pulping process: kraft, lignosulfonate, soda, and organosolv lignin. Most lignin is produced by the kraft process, which constitutes the largest volume of all lignin types [63]. For example, Liu et al. [64]. fabricated a kraft lignin-based superhydrophobic powder by modifying kraft lignin using 1H, 1H, 2H, 2H-perfluorodecyl-triethoxysilane (PFDTES, Shanghai, China) substitution reactions and formed superhydrophobic coatings by depositing the suspended PFDTES-lignin powder directly onto various substrates. The formed lignin-based coatings exhibited an outstanding water repellency, with a water contact angle of 164.7°, as well as good friction; acid, alkali, and salt resistance; and fairly excellent self-cleaning properties. Yu et al. [65] synthesized micrometer-sized porous oil/water separation materials with a superhydrophobic surface in one step using kraft lignin.

### 4.2. Modification Preparation of Superhydrophobic Surfaces

The preparation of any superhydrophobic surface requires a combination of micro- nano rough structures and low-surface-energy substances. In recent years, a number of physical and chemical methods have been developed for the preparation of superhydrophobic surfaces. These methods can be categorized into one-step processes and multi-step processes.

#### 4.2.1. One-Step Method

**Figure 5:**
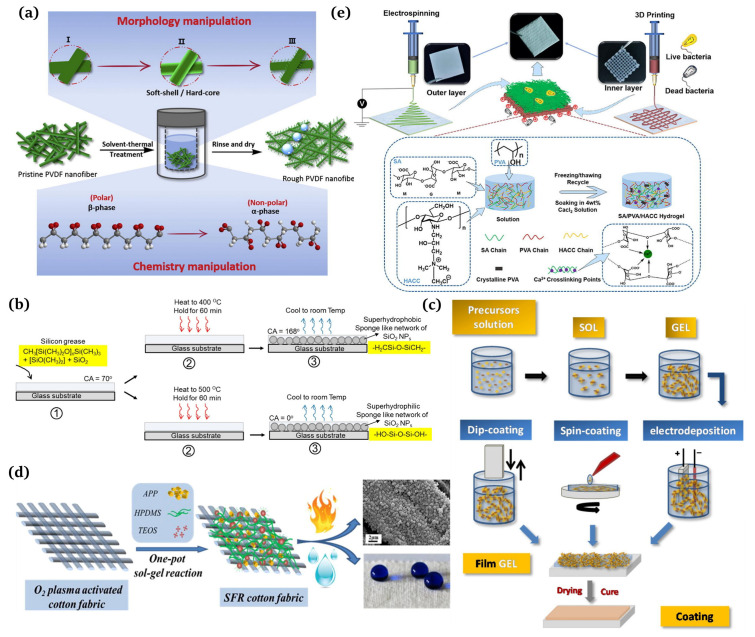
(**a**) Schematic representation of the surface roughness and chemical properties of a one-step manipulated PVDF membrane [66]; (**b**) one-step preparation of transparent superhydrophobic surfaces [67]; (**c**) processing routes for obtaining sol-gel coatings [82]; (**d**) one-pot production of superhydrophobic flame retardant coatings [86]; (**e**) preparation and characterization of double-layer asymmetric dressing through electrospinning and 3D printing [96].

A one-step method refers to completing the surface roughening and chemical modification in one step. Qing et al. [66] published a study detailing a one-step technique for addressing both the surface roughness and surface chemistry of PVDF nanofiber membranes for improved direct contact membrane distillation (DCMD) performances (Figure 5a). Siddiqui et al. [67] prepared self-cleaning transparent superhydrophobic silica-based surfaces using one-step thermal oxidation for silicone grease, with a water contact angle (CA) of 168° and a slip angle (SA) of 2°. Initially, the substrates were lubricated with silicone grease and then underwent one-step thermal oxidation at 400 °C (Figure 5b). Many mature preparation processes have been reported [68], such as sol-gel [69,70], template [71,72], electrospinning [73,74], phase separation [75,76], layer-by-layer assembly [77,78], and etching [79,80]. Several typical preparation methods are introduced here.

##### Sol-Gel

The sol-gel method uses some compounds with high-active chemical ingredients as the precursors. Chemical reactions such as mixing, hydrolysis, and condensation are carried out in the liquid phase to form a sol-gel system. Then, the gel state is shaped on the textile surface using spinning, dip plating, coating, or other methods to obtain the structured rough fabric [81]. Figure 5c demonstrates the operating steps of the sol-gel method [82]. It can form transparent, adhesive metal, or non-metal oxide films on the fabrics, which can be modified to improve the hydrophobicity significantly. Guo et al. [83] obtained superhydrophobic surfaces of polyester sponges, filter cloths, and filter papers using the sol-gel method. Goncalves G et al. [84] fabricated a multi-step nanoengineering process to produce superhydrophobic cellulose nanocomposites. The surface roughness of the cellulose fibers was promoted by adding ${\mathrm{SiO}}_{2}$ particles of different sizes and the surface energy was reduced using chemical modification with two types of fluorosiloxanes. Li et al. [85] successfully manufactured superhydrophobic surfaces using the sol-gel method and utilizing water glass as the starting material. Firstly, such surfaces were formed by dip coating the silica hydrosols prepared by hydrolysis and the condensation of water glass on the cotton substrates. Then, the surface of the silica coating was modified with a non-fluoro compound, hexadecyltrimethoxysilane (HDTMS, Fluka), to obtain a thin film through self-assembly, thus acquiring the superhydrophobic surface with a water contact angle higher than 151°. Lin et al. [86] proposed a facile one-pot gel-sol method to construct superhydrophobic flame retardant (SFR) coatings on cotton fabrics (Figure 5d). XU et al. [87,88,89] developed superhydrophobic modified cotton by coating cotton fabrics using the trimethylsilane-modified impregnation press method. In short, the sol-gel method is simple, easy to operate, inexpensive, and less restrictive on the substrate material, so it has been favored by researchers in the construction of nanostructures.

##### Electrospinning

Recently, nanotechnology has developed rapidly, making nanomaterials a significant research area for scholars. Electrospinning serves as a simple and practical means of preparing nanomaterials and microfine nanostructures, making it a broadly used technology. The preparation of superhydrophobic fiber materials using electrospinning has attracted widespread attention due to its simple technical equipment, low cost, and controllable process. Traditional electrospinning devices mainly consist of three parts: a high-voltage power supply, a liquid supply device, and a fiber collector [90]. Oktay et al. [91,92] obtained electrospun mats which showed a special surface structure. The surface was different from that of water-in-oil emulsion and had a contact angle of 167° with water, exhibiting an excellent self-cleaning function. Zhang et al. [93] demonstrated superhydrophobic polyacrylonitrile (PAN) nanofibrous membrane-implanted Au nanoparticles (NPs) in cavity via a facile electrospinning and consecutive surface modification strategy. Cakir et al. [94] sprayed the spinning solution mixed with polyurethane and silica onto the cotton fabric using electrospinning and achieved a contact angle as high as 154.5° on the fabric. Moatmed et al. [95] fabricated a superhydrophobic/superoleophilic fluid-embedded iron (II, III) oxide (II) with ultrafast oil and water separation properties and converted the nanoparticles into polystyrene nanomaterials using electrospinning. Zhang et al. [96] developed an asymmetric dressing for skin wound repair using electrospinning in conjunction with 3D printing technology (Figure 5e). The outer layer was produced by electrospinning the optimized PCL/PLA (PP) to imitate the epidermis with water repellency and antibacterial properties and with a tensile modulus of 19.69 ± 0.66 MPa. The inner layer was 3D printed from optimized sodium alginate/polyvinyl alcohol/chitosan quaternary ammonium salt (SPH).

##### Etching

**Figure 6:**
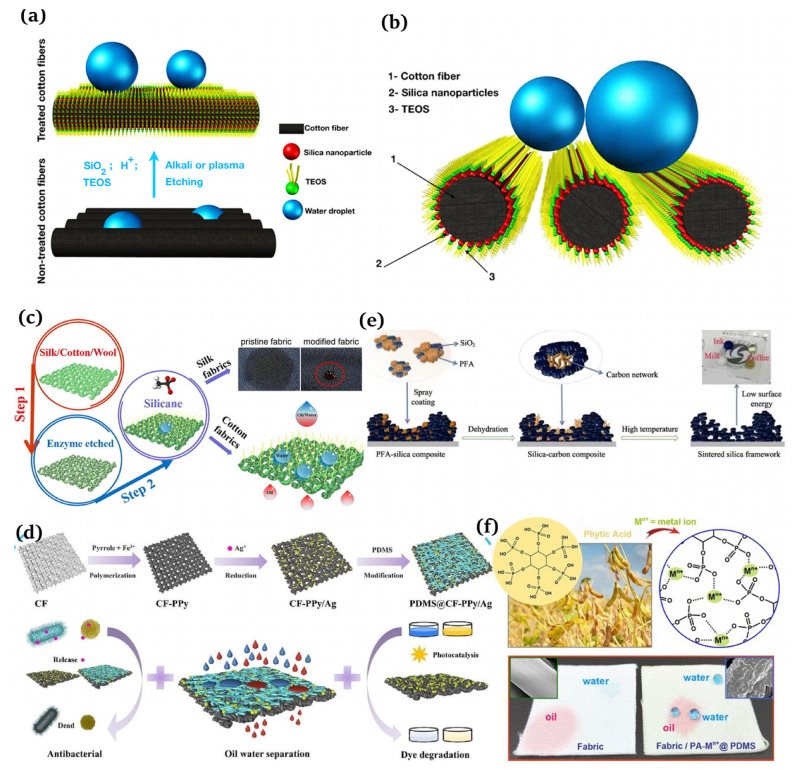
(**a**) Manufacture of robust superhydrophobic cotton fibers usin the dip coating method [103]; (**b**) schematic of a droplet deposited on superhydrophobic cotton [103]; (**c**) fabrication of robust superhydrophobic fabrics using the etching method [104]; (**d**) creation of multifunctional cotton fabrics [106]; (**e**) schematic of a transparent superhydrophobic surface prepared by the phase separation of ${\mathrm{SiO}}_{2}\;$ and PEA [109]; (**f**) design of superhydrophobic fabrics for oil–water separation inspired by nature [110].

The representative etching methods include plasma [97,98], chemical [99,100], and laser [101,102]. Nguyen-Tri et al. [103] employed chemical and plasma etching techniques to combine silica nanoparticles and tetraethyl orthosilicate (TFOS) to produce superhydrophobic fibers, which was simple and fluorine-free (Figure 6a,b). Cheng et al. [104] utilized an environmentally friendly enzymatic etching approach to obtain superhydrophobic textiles, which were modified using methyltrichlorosilane (MTCS, Sigma-Aldrich, St. Louis, MO, USA) at 70 °C through a simple thermal chemical vapor deposition (CVD) protocol (Figure 6c). Wu et al. [105] achieved chemically stable and durable superhydrophobic textiles by using a fluoropolymers (FPs, Jin Tai Auxiliary Chemical Co. Ltd., Pingxiang, China) solution and soaking the coating.

##### Deposition

Deposition refers to the chemical or physical deposition of raw materials onto textiles by means of a layer-by-layer assembly, hydrothermal methods, and vapor phase deposition, forming thin films with rough structures. Zeng et al. [106] successfully integrated various stages of cotton fabric production into a single preparation process, resulting in a multifunctional fabric that was expected to effectively separate oil and water. This innovative approach provided new ideas for the application of multifunctional fabrics in this field (Figure 6d). Li et al. [107] synthesized a carpeted structure of high-purity copper carbon nanotubes (CSCNTs) with a high hydrophobicity and flexibility within 15 min using chemical deposition.

##### Other Methods

In addition, the methods for constructing superhydrophobic rough surfaces also include template, phase separation, and nanoparticle loading. Inspired by lotus leaves, Sun et al. [108] first utilized PDMS to replicate the “micro-nano structures” on the surfaces of lotus leaves, thus obtaining PDMS superhydrophobic surfaces. Zhao et al. [109] employed high-temperature sintered silica and poly(ethylene furoate) (PEA) to form a stable rough structure on the glass surface. After PDMS modification, a superhydrophobic surface with a contact angle greater than 164° and a light transmittance of up to 87% was synthesized (Figure 6e). Zhou et al. [110] proposed a biomimetic approach in their study and utilized metal-coordinated phytic acid aggregates to create a surface roughness on the fabric followed by treatment with PDMS, demonstrating an excellent resistance ability to mechanical abrasion and wear (Figure 6f).

#### 4.2.2. Multi-Step Process

**Figure 7:**
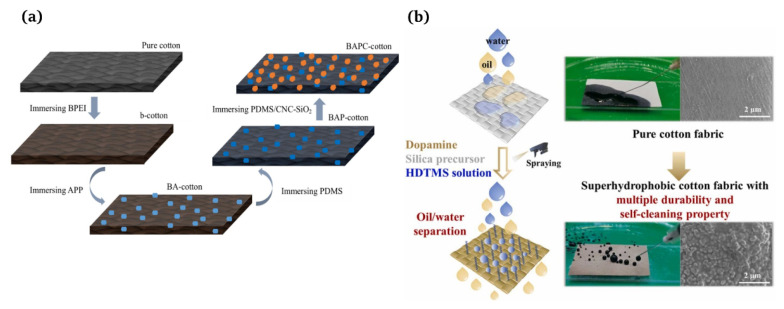
(**a**) Formation mechanism of the flame retardant and superhydrophobic cotton fabric [111]; (**b**) superhydrophobic cotton fabric with improved durability and wearer comfort using spray methods [8].

A multi-step method [3] refers to the fact that after obtaining a rough surface using a subtractive process, the material surfaces need to be modified so the surface can obtain superhydrophobicity using low-surface-energy materials. Huang et al. [111] developed an effective method for producing flame retardant and superhydrophobic cotton fabrics through a multi-step impregnation process. The team demonstrated the excellent flame retardant and hydrophobic properties of this fabric (Figure 7a). Guo et al. [8] fabricated superhydrophobic cotton fabrics with a high durability using a facile spraying method that did not produce waste water and could effectively employ modifiers. Initially, the in situ reaction of dopamine and 2-tetraethoxysilane was used to create a rough surface structure. Then, the hexadecyltrimethoxysilane with long-chain alkyl was firmly bonded to the surface of the cotton fibers to reduce the surface energy (Figure 7b).

## 5. Applications of Biomimetic Superhydrophobic Surfaces

### 5.1. Self-Cleaning

**Figure 8:**
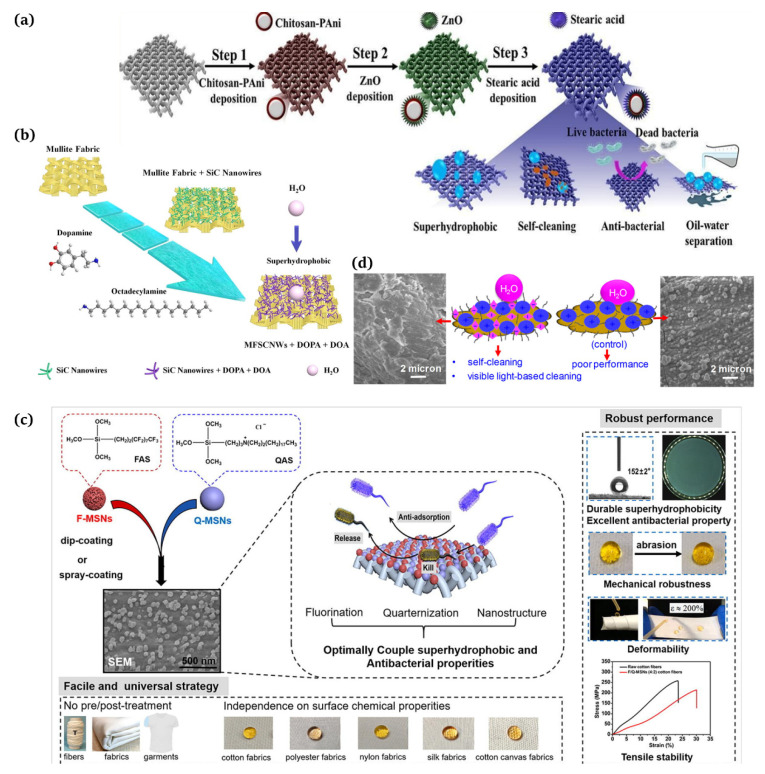
(**a**) Application of self-cleaning, superhydrophobic, and antimicrobial cotton fabrics [115]; (**b**) silicon carbide nanowire modified mullite fabric hierarchical structure applied as a stable and self-cleaning superhydrophobic material [116]; (**c**) textile coatings with antifouling and antibactericidal properties for medical and daily protection situations [117]; (**d**) employment of self-cleaning cotton fabrics and antifungal/antimicrobial surfaces [118].

At present, there is a great research interest in the development of biomimetic and functionalized textile materials, and the preparation of fabrics with self-cleaning surfaces has been vigorously investigated [112,113]. Self-cleaning fabrics have environmentally friendly, stain resistant, and antimicrobial properties [114]. Suryaprabha et al. [115] used a simple and environmentally friendly method to obtain superhydrophobic cotton fabrics by introducing chitosan-based composite coatings on cotton fabrics (Figure 8a). Liu et al. [116] successfully produced non-fluorinated superhydrophobic mullite fabrics stabilized by the in situ growth modification of silicon carbide (SiC) nanowires, dopamine, and octadecylamine (ODA, Shanghai Siyu Chemical Technology Co., Ltd., Shanghai, China) (Figure 8b).

### 5.2. Biomedical Applications

There is a huge potential in medical and general protective applications for fabric coatings that combine the antifouling function of a superhydrophobic surface with the antimicrobial activity of surface-bound antimicrobials. Ye et al. [117] developed a straightforward and versatile method to combine superhydrophobic and antibacterial properties effectively on diverse textiles through a single-step dip coating or spray coating process (Figure 8c). Biswas et al. [118] presented a surface modification approach that produced self-cleaning superhydrophobic cotton employing silica nanoparticles, N,F-doped TiO_2_ nanoparticles, and octadecyltrimethoxysilane (Figure 8d). Ibili et al. [119] proposed a new strategy for fabricating multifunctional textiles featuring superhydrophobic and antibacterial nanoparticles for medical purposes via a one-step electrospraying method.

### 5.3. Functional Textiles

#### 5.3.1. Water Resistant Fabric

**Figure 9:**
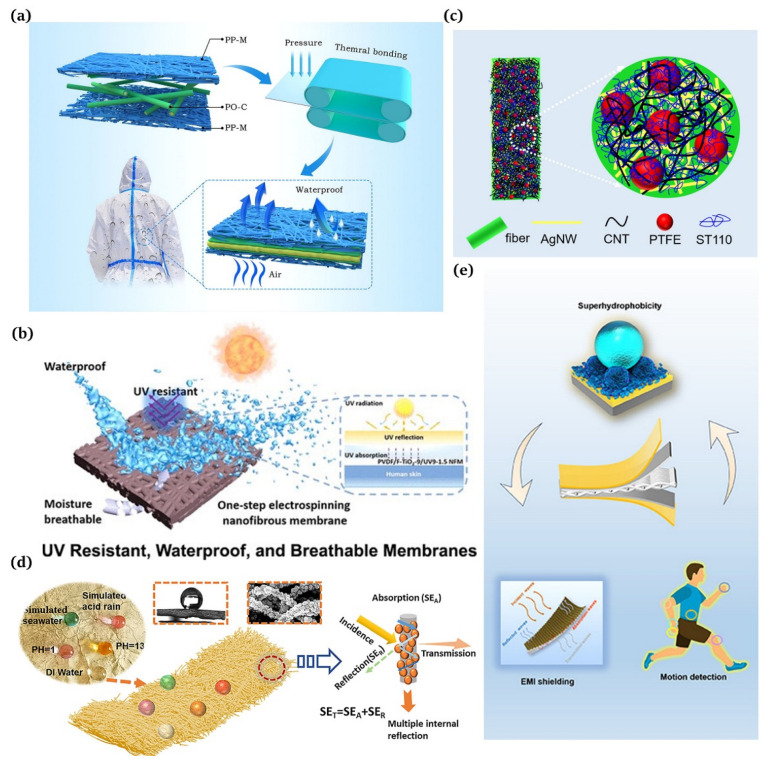
(**a**) Applications of three-layer laminated polyolefin microfiber fabrics [120]; (**b**) wearable smart membranes as a functional core layer in fabrics [121]; (**c**) silver nanowire (AgNW) networks and superhydrophobic coatings for commercial textile products [122]; (**d**) employment of superhydrophobic and corrosion resistant electrospinning hybrid membranes for efficient electromagnetic interference shielding [123]; (**e**) superhydrophobic e-textiles with an Ag-EGaIn conductive layer for motion detection and EMI shielding [124].

Waterproof and breathable materials, as their name implies, hinder liquid water from entering while letting gaseous molecules pass through, displaying their ability of repelling water and moisture. There are two classes of waterproof breathable materials based on their moisture permeability mechanisms: hydrophobic microporous and hydrophilic non-porous materials. One exemplary product of hydrophobic microporous membranes is the polytetrafluoroethylene (PTFE) film produced by Gore-Tex using the double-stretching technique. This film boasts exceptional waterproofing and windproofing capabilities, as well as impressive heat resistance and chemical stability. Moreover, it can be fashioned into outdoor apparel, including mountaineering clothing and punching jackets. Zhang et al. [120] developed a new three-layer polyolefin composite fabric with remarkable waterproof and breathable qualities using the thermal bonding process, which demonstrated high strength and effective filtration properties (Figure 9a). Liu et al. [121] employed a one-step electrospinning technique to conveniently fabricate eco-friendly waterproof and breathable nanofiber membranes with a high UV resistance (Figure 9b).

#### 5.3.2. Wearable Electronoas/Electromagnetic Interference

For the last few years, there has been widespread use of electromagnetic shielding fabrics due to their ability to protect individuals from electromagnetic radiation sources [50]. Jia et al. [122] designed a robust superhydrophobic conductive fabric for efficient EMI shielding (Figure 9c). Yang et al. [123] created superhydrophobic electromagnetic shielding materials by chemically depositing silver nanoclusters on electrospinning polymer nanofibers followed by stearic acid (SA) modification (Figure 9d). Sun et al. [124] present a straightforward and effective method for integrating elastic e-textiles by employing a multi-stage approach (Figure 9e).

### 5.4. Environmental Remediation

#### 5.4.1. Water–Oil Separation

**Figure 10:**
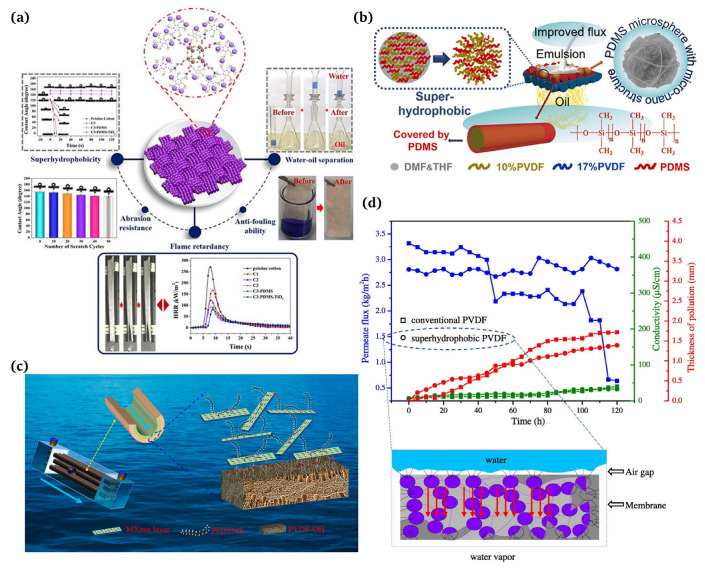
(**a**) Employment of superhydrophobic coatings for water/oil separation [125]; (**b**) applications of superhydrophobic membranes for ultrafast separation of water-in-oil emulsions [126]; (**c**) hydrophobic–hydrophilic MXene/PVDF composite hollow fiber membranes with improved antifouling properties for seawater treatment [130]; (**d**) a new superhydrophobic electrospun PVDF membrane for seawater desalination [131].

Based on the principle of separating the mixtures of two solutions with different surface tensions, special wettable textiles can be classified into two types, namely filter membranes and absorbent materials [50]. Guo et al. [125] used a simple two-step spray method to create a flame retardant and superhydrophobic coating that was deposited on cotton fabrics (Figure 10a). Cheng et al. [126] invented a simple method of coaxial electrospinning to produce superhydrophobic membranes for the ultrafast separation of water-in-oil emulsions (Figure 10b). Abu-Thabit et al. [127] proposed a facile, eco-friendly, and efficient method for fabricating superhydrophobic polyacrylonitrile (PAN) non-woven fabrics (NWF) for the separation of oil/water mixtures by employing adsorption and filtration strategies. The superhydrophobic NWF demonstrated a commendable oil absorption capacity, high flux permeability, and outstanding separation efficiency. Saeed et al. [128] invented a facile dip coating approach for obtaining a superhydrophobic oil/water separation membrane by anchoring vinyltris(2-methoxyethoxysilane) onto the surface of the cotton fabric.

#### 5.4.2. Seawater Desalination

Membrane distillation (MD) is a promising seawater desalination technology due to its potential for processing high-salinity water and being driven by low-grade or waste heat. However, a low vapor flux and fouling problems limit the practical application of MD membranes. Recently, there has been increasing attention in the development of novel MD membrane materials with enhanced hydrophobicity to improve the desalination efficiency [129]. Zhang et al. [130] succeeded in producing a hydrophobic–hydrophilic MXene/PVDF composite hollow fiber membrane (PVDF-MP) with considerable anti-wetting and antifouling performances via a chemical grafting method (Figure 10c). Su et al. [131] designed a new superhydrophobic electrospun poly (vinylidene difluoride) membrane and applied it to MD desalination (Figure 10d).

## 6. Conclusions and Perspectives

In modern manufacturing, textiles have not been confined to clothing in people’s perception. They have been widely applied in fields such as aerospace, industrial safety products, interior decoration, etc. Functionalization and biomimetic technologies have received increased attention in modern textiles. Modified superhydrophobic textiles have gained popularity due to their excellent antifouling and waterproofing performances. Therefore, modifying superhydrophobic textiles has become a crucial focus of functional textile research. Currently, the primary methods for textile modification involve constructing “micro-nanostructures” on the textile surface and coating textile surfaces with chemical substances that can reduce the surface energy. The applications of superhydrophobic fabrics in medical, aerospace, and environmental governance fields have been widely investigated. Despite the recent development of various materials and methods for generating superhydrophobic surfaces, several issues still exist. (1) At present, many techniques for creating superhydrophobic surfaces are complex in terms of their processes. (2) The substances utilized to reduce the surface energy, such as organosilicons and fluorine-containing compounds, are both costly and harmful to the environment. (3) Although micro-nano structures are integral for ensuring hydrophobicity, they fail to take into account the mechanical properties of the materials. Henceforth, researchers should direct their attention towards enhancing the mechanical stability and self-repairing characteristics of superhydrophobic surfaces, while concurrently endeavoring to decrease costs, streamline manufacturing processes, and introduce environmentally friendly raw materials. These findings offer a promising prospect for the development of superior superhydrophobic fabrics.

## Data Availability

Not applicable.
